# The Score Model Containing Chinese Medicine Syndrome Element of Blood Stasis Presented a Better Performance Compared to APRI and FIB-4 in Diagnosing Advanced Fibrosis in Patients with Chronic Hepatitis B

**DOI:** 10.1155/2016/3743427

**Published:** 2016-01-20

**Authors:** Xiao-Ling Chi, Mei-Jie Shi, Huan-Ming Xiao, Yu-Bao Xie, Gao-Shu Cai

**Affiliations:** Hepatology Department, Guangdong Provincial Hospital of Chinese Medicine, Guangzhou, Guangdong 510120, China

## Abstract

This study aims to explore a useful noninvasive assessment containing TCM syndrome elements for liver fibrosis in CHB patients. The demographic, clinical, and pathological data were retrospectively collected from 709 CHB patients who had ALT less than 2 times the upper limit of normal from April 2009 to October 2012. Logistical regression and area under receiver-operator curve (AUROC) were used to determine the diagnostic performances of simple tests for advanced fibrosis (Scheuer stage, F ≥ 3). Results showed that the most common TCM syndrome element observed in this CHB population was dampness and Qi stagnation, followed by blood stasis, by heat, and less by Qi deficiency and Yin deficiency. The logistical regression analysis identified AST ≥ 35 IU/L, PLT ≤ 161 × 10^9^/L, and TCM syndrome element of blood stasis as the independent risk factors for advanced fibrosis. Therefore, a score model containing these three factors was established and tested. The score model containing blood stasis resulted in a higher AUC (AUC = 0.936) compared with APRI (AUC = 0.731) and FIB-4 (AUC = 0.709). The study suggested that the score model containing TCM syndrome element of blood stasis could be used as a useful diagnostic tool for advanced fibrosis in CHB patients and presented a better performance compared to APRI and FIB-4.

## 1. Background

Hepatitis B virus (HBV) carriers and chronic hepatitis B (CHB) patients with minimally elevated alanine aminotransferase (ALT) have been increasingly attended in recent years. More and more evidences showed that patients with ALT less than 2 times the upper limit of normal (<2ULN) could still eventually develop clinical complications [1]. Several studies [2, 3] observed significant fibrosis in CHB patients with minimally elevated ALT, even in patients with normal ALT. It is well known that advanced liver fibrosis results in cirrhosis, liver failure, and portal hypertension and often requires liver transplantation. So, it is quite important to identify the patients with higher risk of developing advanced fibrosis and then to diagnose and intervene early. As the “gold standard” to diagnose and determine the stages of fibrosis in CHB patients, however, the wide application of liver biopsy was limited due to its invasive pattern, risks, and expensive cost. The available noninvasive diagnostic assessments including APRI, FIB-4, and FibroTest were also less optimal, although they have been increasingly accepted in clinical practice. Based on our clinical experiences in diagnosis and treatment with Traditional Chinese Medicine (TCM) in CHB patients with fibrosis, TCM syndrome elements would improve the diagnosis performance when combined with the markers with confirmed diagnostic effects in these patients.

In a word, previous studies in domain of modern medicine have identified several useful markers in predicting liver fibrosis in CHB patients [2, 4–6]; however, there were few studies that evaluated the TCM factors in predicting and diagnosing liver fibrosis in CHB patients with ALT < 2ULN in the domain of TCM sciences. So, we conducted this study in CHB patients with ALT < 2ULN to identify the characteristics of TCM syndromes associated with the developing of liver fibrosis and to explore a useful noninvasive assessment that contained the TCM syndrome elements for diagnosing liver fibrosis in CHB patients.

## 2. Methods

### 2.1. Subjects

As an earlier stage of a study registered in 2014 evaluating the blocking effects of liver fibrosis with TCM treatment in CHB patients with ALT < 2ULN, the demographic, clinical, and pathological data were retrospectively collected from CHB patients who had ALT < 2ULN and performed liver biopsy in the Guangdong Provincial Hospital of Chinese Medicine between April 2009 and October 2012. The diagnoses of CHB and live fibrosis were determined according to the criteria described in Guideline of Prevention and Treatment for Chronic Hepatitis B (2010 version) [7] and Guideline for Diagnostic and Treatment of Hepatic Fibrosis [8], and the TCM syndrome elements were determined according to Standards of Traditional Chinese Medicine syndrome differentiation for viral hepatitis (trial implementation) [9] and the diagnostic standard of TCM syndrome elements for CHB patients [10].

All patients who meet the following inclusion criteria could be enrolled: (1) those aged from 18 to 65 years, (2) those having a diagnosis of CHB, (3) HBsAg being positive and ALT being less than 2ULN for at least 6 months prior to the study, and (4) patients with diagnosis of single or combined TCM syndrome (e.g., dampness or heat or blood stasis). Patients with any of the following conditions were excluded from the study: (1) coinfection with another hepatitis virus or human immunodeficiency virus; (2) concomitant liver and gallbladder diseases which can interfere in the evaluation of risk factors including evident advanced primary biliary cirrhosis, autoimmune hepatitis, decompensated cirrhosis, severe hepatitis, or hepatic carcinoma and a history of excessive alcohol consumption (20 g per day for female and 30 g per day for male); (3) concomitant uncontrolled serious heart, kidney, lung, endocrine, blood, metabolic, or gastrointestinal primary disease or mental illness; (4) patients who received any antifibrotic treatments within 6 months prior to the enrollment. The study has been approved by the ethics committee of Guangdong Provincial Hospital of Chinese Medicine, and written informed consent was obtained from all patients during outpatient follow-up or by telephone and letter follow-up before this study.

### 2.2. Clinical and Laboratory Assessment

The demographic, clinical, and laboratory data were collected at the time of liver biopsy, including age, gender, levels of ALT, aspartate aminotransferase (AST), g-glutamyl transpeptidase (GGT), albumin, platelet count, and viral parameters. Serum HBsAg was measured by electrochemical immunoassay (Elecsys 2010; Roche Diagnostics, Mannheim, Germany). Serum HBV DNA was measured with a lower limit of detection of 500 IU/mL (ABI 7300, Applied Biosystems Inc., USA).

### 2.3. Histological Assessment

Liver biopsy was performed using 16G MAXCO needles (Bard Co., Murray Hill, NJ, USA). The specimens were fixed in 10% formalin, paraffin-embedded, and stained with hematoxylin and eosin in turn. A minimum of 15 mm length of liver tissue and at least five portal tracts were required for diagnosis. Fibrosis stage was determined according to Scheuer's classification: F0, no fibrosis; F1, enlarged fibrotic portal tracts; F2, periportal or portal-portal septa, but intact architecture; F3, fibrosis with architectural distortion, but no obvious cirrhosis; and F4, cirrhosis. Liver fibrosis beyond the portal tract (stage F3 or F4) was considered advanced fibrosis [11].

### 2.4. Data Analysis

Continuous variables were expressed as mean ± standard deviation, or median and interquartile range, and were compared with Student* t*-test or nonparameter test (Mann-Whitney) as appropriate. Zero and one were used to express binary enumeration data and frequency was counted (such as the measurement of TCM variables). Categorical parameters among groups were compared by chi-squared test. Univariate analysis was performed to identify the variables associated with advanced fibrosis. Then, a multivariate logistic regression analysis that contained these significant variables (*P* < 0.05) identified by univariate analysis was performed to determine the risk factors for advanced fibrosis. Area under receiver-operator curve (AUROC) was used to calculate the diagnostic accuracy of the score model established in this study as well as aspartate aminotransferase to platelet ratio index (APRI) and fibrosis index based on the 4 factors (FIB-4) in diagnosing advanced fibrosis. Delong's test was used to compare the AUC of these three noninvasive tests. Statistical significance was taken as *P* < 0.05 at two sides. All statistical analysis was performed using SPSS software version 16.0 (SPSS Inc., Chicago, IL, USA).

## 3. Results and Discussion

### 3.1. Results

#### 3.1.1. Characteristics of the 709 CHB Patients with ALT < 2ULN

The basic demographic and clinical characteristics of the 709 enrolled CHB patients were showed in Table 1. Among the 709 patients, 436 (61.49%) were patients with normal ALT and 273 (38.51%) were patients with minimally elevated ALT (40 < ALT < 80); 109 (15.4%) patients had advanced inflammation activity and 232 (32.7%) patients had advanced fibrosis. The demographic data showed that there were more men than women and the median age was 39 years in all patients. The patients in minimally elevated ALT group had significantly higher levels of ALT, AST, and GGT, serum level of HBV DNA, grade of inflammation activity, and the percentage of HBeAg (+) patients than patients in normal ALT group (all of *P* < 0.001, except *P* = 0.018 for inflammation activity). However, no significant difference was found on the stage of fibrosis between these two groups (*P* > 0.05).

#### 3.1.2. Features of TCM Syndrome Elements Distribution in 709 CHB Patients with ALT < 2ULN

As shown in Table 2, among the TCM syndrome elements of CHB patients, dampness and Qi stagnation had the highest frequency, followed by blood stasis, by heat, and less by Qi deficiency and Yin deficiency. Almost all patients had a combined syndrome, and only 29 (4.09%) patients had single syndrome in these 709 CHB patients with ALT < 2ULN.

#### 3.1.3. Univariate and Multivariate Analysis of Factors Associated with Advanced Fibrosis

Compared with the patients without advanced fibrosis (*n* = 477), CHB patients with advanced fibrosis (*n* = 232) had a significant higher percentage of male patients (73.7% versus 66.0%, *P* = 0.039) and higher levels of age (42.2 versus 37.9 years, *P* < 0.001), AST (34.3 versus 27.9 IU/L, *P* < 0.001), and GGT (47.4 versus 27.3 IU/L, *P* < 0.001) but lower platelet counts (161.7 versus 203.7 × 10^9^/L, *P* < 0.001) and HBV DNA (5.08 versus 5.55 log_10_ IU/mL). Moreover, all the CHB patients with advanced fibrosis showed higher frequency of the TCM syndrome elements of blood stasis and Qi deficiency (*P* < 0.001) (Table 3) and lower frequency of the TCM syndrome element of heat.

The univariate analysis identified the variables including male, age, AST, PLT, GGT, HBV DNA, and TCM syndrome elements of blood stasis, Qi deficiency, and heat as the factors associated with advanced fibrosis. However, further multivariate logistic regression analysis including all above factors revealed that only increased serum AST level, decreased PLT count, and TCM syndrome element of blood stasis were associated with advanced fibrosis. Based on the measurements of mean level of AST and PLT in patients with advanced fibrosis, we selected AST ≥ 35 IU/L, PLT ≤ 161 × 10^9^/L, and TCM syndrome element of blood stasis as the independent factors for advanced fibrosis with regression coefficients of 0.816, 1.379, and 5.457, respectively (Table 4). Then as shown in Table 5, each factor was assigned a score based on regression coefficients to establish a simplified score model to serve as a noninvasive assessment for diagnosing advanced fibrosis.

#### 3.1.4. Comparison of Diagnostic Accuracy of the Score Model Containing Blood Stasis, APRI, and FIB-4 in Advanced Fibrosis

**Figure 1:**
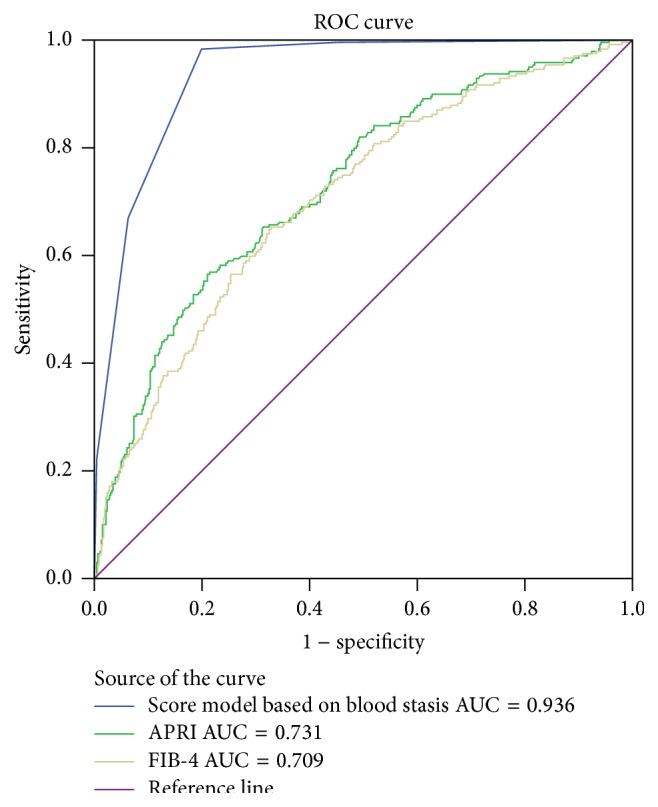
Comparison of predictive models for advanced fibrosis with ROC analysis.

As shown in Figure 1, the score model containing blood stasis resulted in a higher AUC (AUC = 0.936) compared with APRI (AUC = 0.731) and FIB-4 (AUC = 0.709) and showed a statistically better performance than APRI and FIB-4 (both *P* < 0.05). Moreover, at the cutoff point of 2 determined by ROC analysis, sensitivity and specificity of the score model established in our study were 98.3% and 80.0%, and positive predictive value and negative predictive value were 71.5% and 98.9%, respectively, at the maximum Youden index when used to diagnose advanced fibrosis. A score of 0-1 indicated patients at lower risk of advanced fibrosis (0–1.1%), whereas a score of 2–4 indicated patients at higher risk (71.5–96.4%).

### 3.2. Discussion

In CHB patients, liver biopsy is currently used as the “gold standard” to diagnose and determine the stages of fibrosis. However, its wide application was limited due to its invasive pattern, risks, and expensive cost. Therefore, there is a great need of some noninvasive assessments which can more accurately identify patients with advanced fibrosis. The available noninvasive assessments including APRI, FIB-4, and FibroTest have gained increasing acceptance in clinical practice [12]. However, there are still some limits of these noninvasive assessments [13]. Moreover, there were few studies focused on the association of TCM terms with the development of fibrosis in domain of TCM. So, we conducted this study to explore a useful noninvasive assessment based on TCM syndrome elements for liver fibrosis in CHB patients.

TCM syndromes can reflect the cause, focus, and nature of a disease and the relationship between evil Qi and vital energy. In this study, we observed that among the TCM syndrome elements of CHB patients dampness and Qi stagnation had the highest frequency, followed by blood stasis, by heat, and less by Qi deficiency and Yin deficiency. It suggests that the etiology and pathogenesis of CHB are very complex. The pathogenic factors were mainly dampness, Qi stagnation, heat, and blood stasis, same as the results from the study by Xie et al. [14]. However, with multivariate logistic regression analysis, only the TCM syndrome element of blood stasis was identified to be associated with advanced fibrosis in our study. It means that patients with blood stasis are susceptible to developing advanced fibrosis in CHB patients. So, the TCM therapy of “activating blood and removing stasis” might decrease the risk of advanced fibrosis in CHB patients. This was also similar to the conclusion previously reported [15, 16]. The study by Tang et al. [15] proposed that the severity of liver-blood stasis might reflect the severity of live fibrosis to a certain extent. Liu et al. [16] in their study demonstrated the effects of different “activating blood” therapies in improving clinical symptoms of chronic hepatitis B liver fibrosis patients and their serum biochemical indicators including A/G level, hyaluronic acid, and laminin. In the future, more studies are needed to further explore the pathogenesis of blood stasis on the development of liver fibrosis and the efficacy of “activating blood and removing stasis” to decrease the risk of liver fibrosis.

In addition to the TCM syndrome element of blood stasis, the multivariate analysis also identified the markers of AST ≥ 35 IU/L, PLT ≤ 161 × 10^9^/L as the independent risk factors for advanced fibrosis. As planned, a risk score model based on the detected factors was established. The score model containing above three risk factors resulted in a higher AUC (AUC = 0.936), compared with APRI (AUC = 0.731) and FIB-4 (AUC = 0.709). The more important thing was that our score model had high sensitivity and specificity of 98.3% and 80.0%, respectively. It suggested that our score model might become a more useful noninvasive assessment for advanced fibrosis compared to APRI and FIB-4. However, a potential limitation of this study was that it was a retrospective, single-center study. Stratification of continuous variables as well as conversion of the regression coefficients to score values although necessary for clinical practicality could be assumed to have resulted in a loss of information and decreased model accuracy. Therefore, further large and prospective studies with long-term follow-up should be conducted to confirm the diagnostic accuracy of our score model containing the TCM syndrome element of blood stasis for the prediction of advanced fibrosis.

## 4. Conclusions

This study clearly demonstrated that the pathogenic factors of CHB patients were mainly dampness, Qi stagnation, heat, and blood stasis. Moreover, the score model containing TCM syndrome of blood stasis, serum ALT, and PLT could be used as a useful noninvasive assessment for advanced fibrosis. However, the diagnostic and predicting role of our noninvasive assessment containing TCM syndrome element of blood stasis for advanced fibrosis will be confirmed in prospective study in the future.

## Figures and Tables

**Table 1:** Characteristics of 709 CHB patients with ALT < 2ULN.

Variables	All patients	0 < ALT ≤ 40	40 < ALT ≤ 80	*P* value
	(*n* = 709)	(*n* = 436)	(*n* = 273)	
Demographic characteristics				
Age (years), median (IQR)	39.0 (31.5–46.0)	39.0 (32.0–45.0)	38.0 (31.0–47.0)	0.823
Males, *n* (%)	486 (68.5%)	273 (62.6%)	213 (78%)	<0.001
Laboratory data				
ALT (IU/L), median (IQR)	35.0 (22.0–47.0)	25.0 (18.0–33.0)	51.0 (45.0–64.0)	<0.001
AST (IU/L), median (IQR)	26.0 (21.0–36.0)	22.6 (19.0–27.0)	36.0 (30.0–43.0)	<0.001
ALB (g/L), median (IQR)	44.1 (42.0–46.4)	43.9 (42.0–46.1)	44.4 (41.95–46.85)	0.280
GGT (IU/L), median (IQR)	24.0 (17.5–38.0)	21.0 (15.0–33.0)	31.0 (22.0–45.0)	<0.001
PLT (10^9^/L), median (IQR)	186 (152.0–224.0)	187 (151.0–223.0)	185 (152.0–225.0)	0.828
HBeAg (+), *n* (%)	331 (46.7%)	178 (40.8%)	153 (56.0%)	<0.001
HBV DNA (log_10_ IU/mL), median (IQR)	5.49 (3.62–7.21)	4.72 (3.24–6.79)	6.29 (4.98–7.59)	<0.001
Liver histology				
Inflammation activity, *n* (%)				
G0–2, *n* (%)	600 (84.6%)	380 (63.3%)	220 (36.7%)	0.018
G3-4, *n* (%)	109 (15.4%)	56 (51.4%)	53 (48.6%)	
Fibrosis (%)				
F0–2, *n* (%)	477 (67.3%)	296 (62.1%)	181 (37.9%)	0.661
F3-4, *n* (%)	232 (32.7%)	140 (60.3%)	92 (39.7%)	

ALT, alanine aminotransferase; AST, aspartate aminotransferase; ALB, albumin; GGT, g-glutamyl transpeptidase; PLT, platelet; HBeAg: hepatitis B e antigen; IQR, interquartile range.

**Table 2:** Distribution of TCM syndrome elements in CHB patients with ALT < 2ULN.

TCM syndrome elements	Dampness	Qi stagnation	Blood stasis	Heat	Qi deficiency	Yin deficiency
*N*	697	651	311	304	173	7
%	98.3%	91.8%	43.9%	42.9%	24.4%	1.0%

TCM, Traditional Chinese Medicine; CHB, chronic hepatitis B.

**Table 3:** Comparison between the patients with advanced fibrosis and without advanced fibrosis.

Variables	Patients without advanced fibrosis	Patients with advanced fibrosis	*P* value
	(*n* = 477)	(*n* = 232)	
Demographic characteristics			
Age (years) (*X* ± SD)	37.9 ± 9.7	42.2 ± 10.6	<0.001
Males, *n* (%)	315 (66.0%)	171 (73.7%)	0.039
Laboratory data			
ALT (IU/L) (*X* ± SD)	35.9 ± 17.8	37.7 ± 16.9	0.205
AST (IU/L) (*X* ± SD)	27.9 ± 11.9	34.3 ± 17.9	<0.001
ALB (g/L) (*X* ± SD)	44.4 ± 3.9	44.6 ± 26.1	0.819
GGT (IU/L) (*X* ± SD)	27.3 ± 19.8	47.4 ± 55.9	<0.001
PLT (10^9^/L) (*X* ± SD)	203.7 ± 53.2	161.7 ± 50.0	<0.001
HBeAg (+) patients, *n* (%)	238 (49.9%)	93 (40.1%)	0.014
HBV DNA (log_10_ IU/mL, *X* ± SD)	5.55 ± 2.09	5.08 ± 1.75	0.004
TCM syndrome elements			
Dampness	471 (98.7%)	226 (97.4%)	0.198
Qi stagnation	438 (91.8%)	213 (91.8%)	0.780
Blood stasis	84 (17.6%)	227 (97.8%)	<0.001
Heat	269 (56.4%)	35 (15.1%)	<0.001
Qi deficiency	101 (21.2%)	72 (31.0%)	0.004
Yin deficiency	4 (0.8%)	3 (1.3%)	0.566

ALT, alanine aminotransferase; AST, aspartate aminotransferase; ALB, albumin; GGT, g-glutamyl transpeptidase; PLT, platelet; HBeAg, hepatitis B e antigen; TCM, Traditional Chinese Medicine.

**Table 4:** The variables associated with advanced fibrosis identified by multivariate logistic regression.

Variables	Regression coefficient	SE	Wald	OR	*P* value
AST ≥ 35 IU/L	0.816	0.317	6.65	2.26	0.010
PLT ≤ 161 × 10e^9^/L	1.379	0.295	21.92	3.97	<0.001
With TCM syndrome element of blood stasis	5.457	0.480	129.3	234.4	<0.001

AST, aspartate aminotransferase; PLT, platelet; TCM, Traditional Chinese Medicine.

**Table 5:** Regression coefficients and the assigned score of factors associated with advanced fibrosis.

Risk factors	Regression coefficient	*P* value	Risk score
AST ≥ 35 IU/L	0.816	0.010	1
PLT ≤ 161 × 10^9^/L	1.379	<0.001	1
TCM syndrome element of blood stasis	5.457	<0.001	2

AST, aspartate aminotransferase; PLT, platelet; TCM, Traditional Chinese Medicine.

## Abbreviations

CHB: Chronic hepatitis B
TCM: Traditional Chinese Medicine
HBV: Hepatitis B virus
ALT: Alanine aminotransferase
<2ULN: Not more than 2 times the upper limit of normal
AST: Aspartate aminotransferase
GGT: g-glutamyl transpeptidase
PLT: Platelet
ALB: Albumin
GLB: Globulin
HBeAg: Hepatitis B e antigen
APRI: AST to platelet ratio index
FIB-4: Fibrosis index based on the 4 factors.
